# Mutation patterns of resistance genes for macrolides, aminoglycosides, and rifampicin in nontuberculous mycobacteria isolates from Kenya

**DOI:** 10.12688/f1000research.124002.3

**Published:** 2022-12-05

**Authors:** Zakayo Mwangi, Grace Naeku, Marianne Mureithi, Frank Onyambu, Wallace Bulimo

**Affiliations:** 1 Department of Medical Laboratory Science, Meru University of Science and Technology, Meru, Meru, 972, Kenya; 2 Department of Medical Microbiology and Immunology, University of Nairobi, Nairobi, Nairobi, 30197, Kenya; 3 Molecular and Infectious Disease Research Laboratory, University of Nairobi, Nairobi, Nairobi, 30197, Kenya; 4 Centre for Molecular Biosciences and Genomics, Centre for Molecular Biosciences and Genomics, Nairobi, Nairobi, 29408, Kenya; 5 Kenya Medical Research Institute, Kenya Medical Research Institute, Nairobi, Nairobi, 54840, Kenya

**Keywords:** Nontuberculous mycobacteria, slow-growing mycobacteria, rapid-growing mycobacteria, macrolides, aminoglycosides, rifampicin

## Abstract

Background: Nontuberculous mycobacteria (NTM) treatment constitutes a macrolide-based antibiotic regimen in combination with aminoglycosides for Rapid-Growing mycobacteria (RGM), and rifampicin for Slow-Growing mycobacteria (SGM). Mutations in the anti-NTM drug target regions promote NTM evolution to mutant strains that are insusceptible to NTM drugs leading to treatment failure. We, therefore, described the mutation patterns of anti-NTM drug target genes including *rrl*, *rrs*, and *rpoB* in NTM isolates from Kenya.

Methods: We carried out a cross-sectional study that included 122 NTM obtained from the sputum of symptomatic tuberculosis-negative patients in Kenya. All the 122 NTM underwent targeted sequencing of the rrl gene. The 54 RGM were also sequenced for *rrs*, and the 68 SGM were sequenced for *rpoB* genes using ABI 3730XL analyzer. The obtained sequences were aligned to their wild-type reference sequences for each gene using Geneious then mutations were identified. Pearson chi-square at 95% confidence interval tested the association of NTM to mutation patterns for each gene.

Results: Twenty-eight (23%) of the NTM harbored mutations associated with resistance to at least one of the macrolide-based therapy antibiotics. Twelve (10.4%)  NTM comprising 7(58.3%) of RGM and 5(41.7%) of SGM had mutations in the *rrl* gene.

For ten (83.3%) NTM, mutations were found at position 2058, while for two (16.6%) NTM, mutations were found at position 2059. Six (11.1%) of the 54 RGM exhibited mutations in the aminoglycoside target gene *rrs* at location 1408. Ten (14.7%) of the 68 SGM harbored mutations in the *rpoB* gene with 40 percent having mutations at codon 531.

Conclusion: We demonstrated a significant level of mutations associated with drug resistance for macrolides, aminoglycosides, and rifampicin in NTM isolated from symptomatic TB negative patients in Kenya.

## Introduction

Non-tuberculous Mycobacteria (NTM) are a group of about 170 Mycobacteria species that do not include *Mycobacterium tuberculosis* and *Mycobacterium leprae* ( Falkinham, 2017; Koh *et al.*, 2006; Peixoto *et al.*, 2020; Sam *et al.*, 2020; Simons *et al.*, 2011). In mycobacterial culture, NTM varies in their growth characteristics, with rapid growing NTM (RGM) forming visible colonies within seven days of incubation and slow-growing NTM (SGM) taking up to fourty days. SGM include *Mycobacterium avium complex* (MAC), *Mycobacterium chimaera*, *Mycobacterium kansasii*, *Mycobacterium malmoense*, and *Mycobacterium xenopi*, whereas RGM comprise species from the *Mycobacterium abscessus* and *Mycobacterium fortuitum complexes* ( Alffenaar *et al.*, 2021). In human infections, identifying NTM is crucial for determining clinically relevant species and determining the best treatment plan ( Chalmers *et al.*, 2019; Mwangi *et al.*, 2022a).

NTM treatment consists of a macrolide-based antibiotic regimen, such as clarithromycin or azithromycin, combined with other selected antibiotics that work synergistically to disrupt NTM metabolic processes and growth ( Falkinham, 2018). In addition to macrolides, the antibiotic of choice is largely determined by the infecting NTM, its growth rate, and the intricacy of the mycolic acid cell wall ( Goswami *et al.*, 2016). Rifampicin and ethambutol are also used in SGM treatment, while aminoglycosides, cefoxitin, imipenem, or tigecycline are used in RGM treatment ( Brown-Elliott *et al.*, 2012; Saxena *et al.*, 2021). Due to the slow growth of NTM compared to other bacterial infections, antimicrobial combination therapy is strongly recommended in NTM treatment to avoid the development of drug resistance ( Falkinham, 2018; Pharmd *et al.*, 2019).

Anti-mycobacterial drugs attach to their binding sites with a high affinity, preventing the target gene product from functioning normally ( Alffenaar *et al.*, 2021). Changes in the structure of the target regions caused by mutations, on the other hand, interfere with the medications' ability to attach to them, resulting in antibiotic resistance. As a result, determining the antibiotic resistance profile of NTM is critical for determining an effective treatment strategy for a specific NTM infection ( Goswami *et al.*, 2016; Nasiri *et al.*, 2017).

Acquired resistance to anti-NTM drugs develops due to mutations in the NTM drug target regions, subsequently promoting NTM evolution to mutant strains that are insusceptible to anti-NTM drugs ( Huh *et al.*, 2019; Munita & Arias, 2016; Nasiri *et al.*, 2017; Pharmd *et al.*, 2019; Saxena *et al.*, 2021). Prolonged exposure to NTM antibiotics, as seen in the lengthy NTM regimen, sub-optimal administration of anti-NTM drugs, as seen in patients who do not adhere to the regimen or who are lost to follow up, and incorrect prescription for NTM infection due to NTM misdiagnosis all promote mutation in the drug target regions ( Gopalaswamy *et al.*, 2020; Munita & Arias, 2016; Zhou *et al.*, 2020).

Macrolides inhibit protein synthesis by binding to the peptide exit tunnel of ribosomes, hence preventing the growing peptide chain from exiting the peptidyl transferase center of the ribosome ( Hansen *et al.*, 2002). Mutation of the *rrl* gene at positions A2058 and A2059 accounts for 80-100% of the macrolide resistance in NTM. Macrolide resistance can also be conferred by the *erm* (41) gene which encodes a ribosomal methyltransferase that methylates the *rrl* thus blocking the drug-binding site of the macrolide ( Bastian *et al.*, 2011; Huh *et al.*, 2019).

Aminoglycosides inhibit protein synthesis by binding the bacterial 30S ribosomal subunit interfering with bacterial protein translation and leading to cell death ( Saxena *et al.*, 2021). Drug resistance to aminoglycosides is associated with modification in the *rrs* gene mostly observed as point mutation at position 1408 ( Brown-Elliott *et al.*, 2013). In addition, mutations at positions 1406, 1409, and 1491 have been shown to confer resistance to aminoglycosides in some NTM ( Olivier *et al.*, 2017).

Rifampicin is a key drug in treating mycobacterial diseases including those caused by *M. tuberculosis* ( WHO, 2014). It inhibits the synthesis of Ribonucleic acid (RNA) by binding to the ß-subunit of the RNA polymerase that is encoded by *rpoB* gene. Most rifampicin-resistant mycobacteria have mutations occurring in an 81-bp rifampicin resistance determining region (RRDR) within the *rpoB* gene. Mutations in this region account for 95% of rifampicin resistance in mycobacteria. The commonly observed mutations within the RRDR of *rpoB* are often seen at codons 526, and 531 ( Li *et al.*, 2016). *M. kansasii* has also shown mutation conferring resistance to rifampicin at codons 513 and 516, while *MAC* also shows resistance to rifampicin with mutations outside the RRDR at codon 626 (K626T) ( Ramasoota *et al.*, 2006).

Detection of drug resistance in NTM can be carried out by drug susceptibility testing (DST) through broth microdilution, ( Litvinov *et al.*, 2018; Park *et al.*, 2020), sequencing for mutations in the *rrl, rrs* and *rpoB* genes ( Brown-Elliott *et al.*, 2012; Huh *et al.*, 2019; Saxena *et al.*, 2021), or by using GenoType NTM-DR test (Hain, Lifescience, Nehren, Germany) which is a line probe assay (LPA) that detects resistance to macrolides and aminoglycosides ( Bouzinbi *et al.*, 2020).

We, therefore, investigated drug resistance in NTM by describing the mutation patterns in *rrl*, *rrs*, and *rpoB* genes for macrolides, aminoglycosides and rifampicin respectively, in NTM isolated from symptomatic TB negative patients from Kenya.

## Methods

We carried out a cross-sectional study that included 122 NTM identified by mycobacterial culture and *hsp65* targeted sequencing from the sputum of symptomatic TB-negative patients ( Mwangi *et al.*, 2022a). The NTM isolates included 54 RGM and 68 SGM. The samples were obtained from the National Tuberculosis Reference Laboratory (NTRL) in Kenya between January to November 2020. All the 122 NTM underwent targeted sequencing of the *rrl* gene. The 54 RGM were also sequenced for *rrs*, and the 68 SGM were sequenced for *rpoB* genes using ABI 3730XL analyzer (Applied Biosystems, Foster City, California, USA).

## Laboratory procedures

### Sample processing, mycobacterial culture and growth identification

The sputum samples were decontaminated using the N-acetyl-L-cysteine 2% NaOH (NALC-NaOH) procedure, then inoculated into Mycobacteria Growth Indicator Tube (MGIT) and Lowestein-Jenseen (LJ) medias, incubated at 37°C and monitored for growth for up to eight weeks respectively. At the same time, sputum smears were prepared, air dried, heat fixed then fluorochrome stained with auramine O where mycobacteria appeared as bright yellow fluorescent rods when viewed under a light emitting diodes (LED) microscope.

The culture growth in MGIT and LJ underwent the Mtb identification testing using the SD Bioline TB Ag MPT64 assay (capilia) (Standard Diagnostics, Yongin-si, Gyeonggi-do, Republic of Korea) and capilia positive samples were excluded from the study. The capilia negative samples underwent ZN microscopy with presence of AFB indicating a possible NTM.

### DNA extraction

Mycobacterial DNA was extracted from 500 μL of re-suspended colonies using using **Geno**Lyse ^®^ ( *Hain* Lifescience, Nehren, Germany) according to the manufacturer's instructions. Briefly, 100 ul of lysis buffer (A-LYS) buffer was added to each cryovial containing the resuspended colonies and incubated for five minutes at 95 ^o^C after which 100 ul neutralization buffer (A-NB) was added and centrifugation done at 5000G for ten minutes. The supernatant was transferred to a newly labelled cryovial awaiting PCR.

### Conventional PCR, DNA gel electrophoresis and DNA purification

Conventional PCR targeting *rrl, rpoB and rrs* genes of NTM were conducted using the Horse-Power™ Taq DNA Polymerase MasterMix (Canvax, Córdoba, Spain) in a final reaction volume for each gene of 13 μl comprising 6.25 μl of 2X Horse-Power™ Taq DNA Polymerase MasterMix, 2.5 μl DNA template, 0.25 μl of each of both forward and reverse primers ( Table 1) at a final concentration of 10 pmoles, and 3.75 μl of nuclease-free water to make up the reaction volume. The PCR assays were carried out with a Veriti Thermal Cycler (Applied Biosystems, Foster City, CA, USA) ( Table 1). Thermal cycling conditions for *rrl* were as follows: one cycle of 95°C for five minutes, 35 cycles of 95°C for one minute, 55°C for one minute, 72°C for one minute, and a final extension for ten minutes at 72°C. PCR for *rrs* was conducted as follows 95°C for five minutes, 35 cycles of 95°C for one minute, 60°C for one minute, 72°C for one minute, and a final extension for seven minutes at 72°C. PCR for the *rpoB* was conducted as follows: 95°C for five minutes, 35 cycles of 95°C for one minute, 58°C for one minute, 72°C for one minute, and a final extension for seven minutes at 72°C. Amplified products were confirmed on a 1% Agarose gel stained with 4.6 μl SYBR safe DNA stain (Invitrogen, Carlsbad, California, USA), and results were visualized with an UltraViolet gel viewer (Terra Universal, S. Raymond Ave, Fullerton, CA, USA). For the corresponding runs, a positive control containing NTM with drug sensitivity to macrolides, aminoglycosides, and rifampicin was used, as well as a negative control containing drug sensitive NTM were included.

**Table 1.** Nucleotide sequence of primers for *rrl*, *rpoB* and *rrs* genes.

Gene	Primers	Size	Reference
*rrl*	F (5′-TTTAAGCCCCAGTAAACGGC-3′) R (5′-GTCCAGGTTGAGGGAACCTT-3′)	420 bp	( Park *et al.*, 2020)
*rpoB*	F (5′-GGCAAGGTCACCCCGAAGGG-3′) R (5′-AGCGGCTGCTGGGTGATCATC-3′)	723 bp	( Adékambi *et al.*, 2003)
*rrs*	F (5′-AAGGAGGTGATCCAGCCGCA-3′) R (5′-TCCCTTGTGGCCTGTGTGCA-3′)	341 bp	( Kim *et al.*, 2021)

The PCR products were enzymatically purified using ExoSAP IT (Applied Biosystems, Foster City, California, USA). Purification conditions were done at 37°C for fifteen minutes followed by a second incubation at 80°C for fifteen minutes and a final cooling step at 4°C for five minutes.

### *rrl, rpoB and rrs* genes sequencing

The purified amplicons were sequenced in the forward and reverse directions by Sanger sequencing using Big Dye™ Terminator Version 3.1 Cycle Sequencing Kit (Applied Biosystems, Foster City, California, USA) and the forward and reverse primers. The sequencing reaction for each gene was a 10 μl reaction comprising 1.25 μl of Big Dye Terminator, 3 μl of 5× Sequencing Buffer, 1 μl of 1 pmol of the sequencing primer, and 1.5 μl of the PCR product. The reaction volume was made up by adding 3.25 μl of nuclease-free water. The reaction proceeded through 96°C for 1 minute then 25 cycles of 96°C for 10 seconds, 50°C for five seconds, and 60°C for four minutes.

Purification of cycle-sequencing products was done using the BigDye X Terminator™ purification kit following the manufacturer’s instructions (Applied Biosystems, Foster City, California, USA) and purified products were loaded onto the ABI 3730 genetic analyzer (Applied Biosystems, Foster City, California, USA) for capillary electrophoresis.

### Data analysis

The obtained sequences were first assembled into contigs and the consensus sequences aligned to their wild-type reference sequences for each gene using Geneious version 11.0 (Biomatters Ltd, Auckland, New Zealand). Mutations in the drug resistance genes were identified visually. STATA version 14 (StataCorp, College Station, Texas, USA) was used to test the association of NTM species to mutation patterns using Pearson chi-square at 95% confidence interval.

## Results

Our study established that twenty-eight (23%) of NTM harbored mutations associated with resistance to at least one of the macrolide-based therapy antibiotics. One isolate (C47) containing *M. abscessus* had mutations conferring resistance in both *rrl* and *rrs* genes.

The bulk of the isolates with mutations associated with drug resistance originated from the Lake Victoria, Coastal, and Nairobi regions, with six (5%) NTM showing mutations in the *rrl*, *rrs*, or *rpoB* genes (p=0.012). The age group was statistically significant (p=0.012), with isolates from participants aged 21 to 35 years old having the highest (n=10, 35.7%) number of NTM with target gene alterations for *rrl*, *rrs*, and *rpoB* genes. Males accounted for twenty one (76.5%) of the 28 NTM isolates with mutations in the drug target genes and indicated a strong statistical significance (p=0.000). The majority of NTM with these mutations were detected in new (n=8; 28.5%) and TB relapse patients (n=8; 28.5%) patients, which had a significant statistical significance (p=0.001) ( Table 2).

**Table 2.** Sociodemographic characteristics of patients with drug-resistant NTM.

Variables	NTM (n, %)	Genes with mutations associated with drug resistance to NTM			P value
		*rrl* (n, %)	*rrs* (n, %)	*rpoB* (n, %)	
**Regions**					
Mt. Kenya	23(18.9)	2(16.7)	-	-	
North Rift Valley	13(10.7)	1(8.3)	-	-	
South Rift valley	9(7.3)	2(16.7)	-	2(20)	
Lake Victoria	8(6.6)	-	2 (33.3)	4(40)	0.012
Lower Eastern	8(6.6)	1(8.3)	-	2(20)	
Coast	33(27)	2(16.7)	2 (33.3)	2(20)	
Northeastern	4(3.3)	-	-	-	
Nairobi	24(19.6)	4(33.3)	2 (33.3)	-	
Subtotal	122(100)	12 (100)	6 (100)	10(100)	
**Age group**					
<21	5(4)	-	-	-	
21-35	49(40.1)	4(33.3)	1(16.7)	5(50)	
36-50	40(32.8)	5(41.7)	3(50)	1(10)	0.012
51-65	21(17.3)	2(16.7)	2 (33.3)	3(30)	
>65	7(5.8)	1(8.3)	0	1(10	
Subtotal	122(100)	12(100)	6(100)	10(100)	
**Gender**					
Female	32 (27)	4(33.3)	2 (33.3)	1(10)	
Male	90(73)	8(66.7)	4(66.7)	9(90)	0.000
Subtotal	122(100)	12(100)	6(100)	10(100)	
**Type of patient**					
New	33(27)	3(25)	2 (33.3)	3(30)	
Retreatment	21(17)	3(25)	1(16.7)	2(20)	
Relapse	30(25)	2(16.7)	2(33.3)	4(40)	0.001
MDR follow-up	38(31)	4(33.3)	1(16.7)	1(10)	
Subtotal	122(100)	12(100)	6(100)	10(100)	

Mutations in *rrl* gene for the NTM were highly significant with a p value of <0.001. Twelve (10.4%) of NTM comprising ( Table 2) seven (58.3%) of RGM ( Table 3) and five (41.7%) of SGM ( Table 3) had mutations within the *rrl* gene. Point mutation at position 2058 was seen in *M.intracellulare*, *M. abscessus subsp abscessus, M. nebraskense, M. massiliense, M. kumamototense, M.* *heraklionense,* and *M. bourgelatii.* Only for *M. abscessus subsp abscessus* was a mutation at 2059 observed ( Table 3).

**Table 3.** NTM showing mutations associated with macrolide resistance.

Isolate ID	NTM species	Growth rate	Position of mutation in *rrl*	*X ^2^*, p value
C21, C64	*M. intracellulare*	SGM	A2058G	
C31	*M. nebraskense*	SGM	A2058C	
C109	*M. kumamototense*	SGM	A2058T	
C132	*M. heraklionense*	SGM	A2058T	
C36, 63	*M. abscessus subsp abscessus*	RGM	A2058C	87, <0.001
C47	*M. abscessus subsp abscessus*	RGM	A2058G	
C88	*M. massiliense*	RGM	A2058T	
C133	*M. bourgelatii*	RGM	A2058C	
C46, C71	*M. abscessus subsp abscessus*	RGM	A2059G	

Mutation at position A1408G of the *rrs* gene was seen for six (11.1%) of the 54 RGM. The NTM species presenting with these mutations include *M. abscessus subsp abscessus, M. chelonae,* and *M. alsense* ( Table 4). The NTM species had a low likelihood (p=0.06) of developing mutations in *rrs* gene for aminoglycoside resistance.

**Table 4.** Rapid-growing NTM showing mutations associated with aminoglycoside resistance.

Isolate ID	NTM species	Position of mutation in *rrs*	*X* ^2^, p value
C12, C53	*M. chelonae*	A1408G	
C58	*M. alsense*	A1408G	25, 0.06
C46, C47, C70	*M. abscessus subsp abscessus*	A1408G	

A low association (p=0.89) for *rpoB* mutation in SGM was observed. Mutations within codons between 503-533 of the *rpoB* were seen for ten (14.7%) SGM. These SGM included *M. avium subsp avium, M. intracellulare, M. yongonense* and *M. gastri* with mutations at codons 506, 509, 516, 526, and 531 respectively ( Table 5).

**Table 5.** Slow- growing NTM showing mutations associated with rifampicin resistance.

Isolate ID	NTM species	Codons with mutation in *rpoB*	*X* ^2^, p value
C66	*M. avium subsp avium*	S531W	
C78, C94	*M. avium subsp avium*	S531L	
C128	*M. avium subsp avium*	S531Y	26, 0.89
C10, C57, C64, C166	*M. intracellulare*	F506L	
C74	*M. yongonense*	E509H	
C151	*M. gastri*	D516V, H526D, S531F	

## Discussion

Nontuberculous mycobacteria ( NTM) are an important cause of pulmonary disease worldwide, and are being isolated increasingly ( Rivero-Lezcano & Carolina González-Cortés, 2019). They are often mistakenly treated as *M. tuberculosis* in countries devoid of laboratory competence for mycobacterial species differentiation ( Pokam *et al.*, 2022). Recently, there has been a considerable rise in infections caused by NTM ( Saxena *et al.*, 2021). These mycobacteria, which comprise a large and diverse range of species, have developed resistance to most conventional antibiotics, rendering their treatments unsatisfactory ( Brown-Elliott *et al.*, 2012).

This study established that 23% of NTM harbored mutations associated with resistance to at least one of the macrolide-based therapy antibiotics. Regional distribution of NTM with mutations in the target genes had a significant correlation (p=0.012) with the bulk of the isolates originating from the Lake Victoria (n=6, 5%), Coastal (n=6, 5%), and Nairobi (n=6, 5%) regions. The observed regional diversity in NTM harboring mutations in drug target genes across Kenya could be attributed to NTM evolution to evade natural antibiotics secreted as secondary metabolites by nearby environmental bacteria in various geographical landscapes (Moore *et al.*, 2019). The 21-35 years age group had the highest number of NTM isolates presenting with drug resistance associated mutations while males comprised the majority (n=21, 76.5%) of cases with NTM showing these mutations. Drug resistance associated mutations was associated with a history of previous TB infection as seen in the high number of TB relapse cases recorded in this study (n=8; 28.5%, p=0.001). This is a common observation in sub-Saharan Africa given the high incidence *of M. tuberculosis* disease and the frequent misdiagnosis of NTM infection with TB seen in this region ( Aliyu *et al.*, 2013; Hoza *et al.*, 2016; Pokam *et al.*, 2022; Mwangi *et al.*, 2022a).

Despite NTM demonstrating high levels of resistance to a broad range of antibiotics, macrolides including clarithromycin, erythromycin, and azithromycin remain the most effective antibiotic with *>*80% of isolates showing susceptibility ( Ananta *et al.*, 2018). However, some NTM including *MAC* and *M. abscessus* have been associated with increased resistance to macrolides leading to treatment failure ( Saxena *et al.*, 2021). The mechanisms of macrolide resistance have been studied at the molecular level and has consistently demonstrated that 80-100% of phenotypic macrolide resistant clinical isolates contained point mutations at positions A2058 and A2059 in the 23S rRNA gene in NTM ( Huh *et al.*, 2019). Our study demonstrated a similar pattern where *M. abscessus subsp abscessus* (A2058G/C, A2059G) and *M. intracellulare* (A2058G) formed the majority of isolates (58.3%) with mutations in the *rrl* gene. Other NTM presenting with mutations associated with macrolides resistance were *M. nebraskense* (A2058C), *M. massiliense* (A2058T) *, M. kumamototense* (A2058T), *M.* *heraklionense* (A2058T), and *M. bourgelatii* (A2058C) *.* The increased potential for development of these mutations in *MAC* species including *M. intracellulare,* and *M. abscessus* could be attributed to inherent factors such as a high propensity for genetic mutation in the drug target region, and high drug tolerance levels ( Park *et al.*, 2020), environmental factors facilitating the emergence of mutations in the *rrl* and subsequent ease of transmission to humans ( Beverley Cherie Millar, 2019). Other mutations that could confer resistance to macrolides include T2419 in *M. intracellulare* ( Huh *et al.*, 2019). However, this mutation was not demonstrated in the Kenyan isolates of this study.

The commonly used aminoglycosides for the treatment of NTM are amikacin, streptomycin, kanamycin, tobramycin, and streptomycin ( Krause *et al.*, 2016). In our present study, three *M. abscessus subsp abscessus*, two *M. chelonae,* and one *M.* *alsense* presented with a A1408G mutation. Genotypic characteristics in *rrs* that are associated with aminoglycoside resistance usually are in concordance with DST broth microdilution and GenoType NTM-DR assays, implying that mutations within *rrs* are the molecular basis of aminoglycoside resistance in NTM ( Bouzinbi *et al.*, 2020). For instance, a study that selected *in-vitro* aminoglycoside-resistant *M. abscessus* and *M. chelonae* presented an A→G mutation at position 1408 within the *rrs* upon sequencing. This confirms that a single point mutation at 1408 is adequate to confer high-level aminoglycoside resistance ( Nessar *et al.*, 2011). Further, individual mutations at T1406, C1409 and G1491 have also indicated considerable resistance to aminoglycoside in most *M. abscessus* subspecies ( Nessar *et al.*, 2011). Similar to other bacteria, NTM present with low-level aminoglycoside resistance through the production of drug-modifying enzymes including acetyltransferases ( Sanz-García *et al.*, 2019), phosphorylases, adenylates, and methylases which act at various points on the aminoglycoside scaffold making it less potent ( Krause *et al.*, 2016; Munita & Arias, 2016; Sanz-García *et al.*, 2019; Tarashi *et al.*, 2022; Zaragoza Bastida *et al.*, 2017).

Similar to *M. tuberculosis*, resistance to rifampicin in NTM is associated with mutations in the 81 bp RRDR corresponding to codons 503 to 533 of the *rpoB* gene ( Saxena *et al.*, 2021; Zhou *et al.*, 2020). Our study identified ten (11.6%) NTM with mutations occurring at codon 531 in *M. avium,* codon 506 in *M. intracellulare*, 509 in *M. yongonense.* *M. gastri* had amino acid substitutions at positions 516, 526, 531 of the *rpoB* gene. Our findings did not establish mutations outside of the RRDR. However, low-level rifampicin resistance has previously been demonstrated in *M. intracellulare* with mutations occurring outside the RRDR at position N494S ( Park *et al.*, 2020). Broth microdilution analysis which is the gold standard for rifampicin drug resistance testing presents a high minimum inhibition concentration (MIC) for isolates with mutations in the RRDR, hence confirming the role of these mutations in conferring high-level resistance to rifampicin ( Huh *et al.*, 2019). We further demonstrated that *M. gastri* harbored more than one mutation within the RRDR which is a unique attribute observed for *M. kansasii* complex species to which *M.gastri* belongs ( Wu *et al.*, 2018). A similar study obtained rifampicin-resistant *M. kansasii* from clinical isolates and *in vitro* generated mutant, and demonstrated mutations in codons 513, 526, and 531 of *rpoB* which is a common pattern in some SGM and *M. tuberculosis* ( Klein *et al.*, 2001).

We found considerable levels of mutations in the drug target genes for macrolides, aminoglycosides and rifampicin in Kenyan NTM. To guide therapy, both species-level identification and drug resistance testing of NTM should be performed before starting treatment for NTM infection.

## Conclusion

We demonstrated a significant level of mutations associated with drug resistance for macrolides, aminoglycosides and rifampicin in NTM isolated from symptomatic TB-negative patients in Kenya.

*M. abscessus* and *MAC* were the dominant NTM with *rrl* mutations, and most mutations occurred at position 2058. Majority of RGM had mutation at position 1408 of the *rrs* gene, while *rpoB* mutations presented within the RRDR.

### Limitations of the study

This study investigated the acquired mechanisms of drug resistance for NTM. Other intrinsic factors apart from drug target gene mutation could influence sensitivity of NTM to antibiotics.

Despite this limitation, the study documents drug resistance associated mutation patterns of Kenyan NTM for the first time, and advocates for drug resistance testing before commencement of treatment for NTM infection.

### Ethical clearance

This study was approved by Kenyatta National Hospital-University of Nairobi Ethics Review Committee (Ref: KNH-ERC/A/38) on 30 ^th^ January 2020. Waiver for individual informed consent was granted as the study utilized remnant clinical samples and the research posed no greater than minimal risk to the study subjects.

## Data availability

Figshare. Mutation Patterns of Resistance Genes for Macrolides, Aminoglycosides, and Rifampicin in Nontuberculous Mycobacteria Isolates from Kenya. DOI: https://doi.org/10.6084/m9.figshare.20331378.v2 ( Mwangi *et al.*, 2022b).

This project contains the following underlying data:

- Mutation patterns for rrl, rrs, and rpoB genes in NTM

Data are available under the terms of the Creative Commons Attribution 4.0 International license (CC-BY 4.0).

## Author contributions

ZMM - Conceptualization, Data Curation, Formal Analysis, Funding Acquisition, Investigation, Methodology, Writing – Original Draft Preparation, Writing – Review & Editing

GN - Investigation, Writing – Review & Editing

MWM - Supervision, Writing- Review and Editing

FGO - Conceptualization, Methodology, Supervision, Writing- Review and Editing

WDB - Conceptualization, Methodology, Supervision, Writing- Review and Editing
