# Supplementary material for: Mutation patterns of resistance genes for macrolides, aminoglycosides, and rifampicin in non-tuberculous mycobacteria isolates from Kenya
Source: F1000Res. 2023 Apr 3;11:962. Originally published 2022 Aug 19. [Version 5] doi: 10.12688/f1000research.124002.5 (PMC10186554; doi:10.12688/f1000research.124002.5)
Supplement: Peer review history

# Peer review: Reviewer response for version 5

**DOI:** 10.5256/f1000research.146329.r168595

**Published:** 2023-04-04

**Authors:** Clement Likhovole

**Affiliations:** 1 Department of Microbiology, UZIMA University, Kisumu, Kenya

The authors have revised the manuscript as per the recommended changes. The revised manuscript is adequate in form and content

Is the work clearly and accurately presented and does it cite the current literature?

Yes

If applicable, is the statistical analysis and its interpretation appropriate?

Yes

Are all the source data underlying the results available to ensure full reproducibility?

Yes

Is the study design appropriate and is the work technically sound?

Yes

Are the conclusions drawn adequately supported by the results?

Yes

Are sufficient details of methods and analysis provided to allow replication by others?

Yes

Reviewer Expertise:

TB molecular biology and immunology

I confirm that I have read this submission and believe that I have an appropriate level of expertise to confirm that it is of an acceptable scientific standard.

# Response

**Published:** 2023-04-05

**Authors:** Zakayo Mwangi

**Affiliations:** Meru University of Science and Technology, Kenya

Thank you for your approval

# Peer review: Reviewer response for version 4

**DOI:** 10.5256/f1000research.144441.r167243

**Published:** 2023-03-24

**Authors:** Clement Likhovole

**Affiliations:** 1 Department of Microbiology, UZIMA University, Kisumu, Kenya

General comments

 The manuscript addresses an important area in Tuberculosis research particularly analysis of drug resistant mutations that have been linked to treatment failure.

 Abstract:

- The authors to include a hyphen in Non-tuberculous Mycobacterium (NTM).
- Use upper case for the first letters of the following acronyms, SGM and RGM.
- In the results section, percentages should not be in brackets. The proportions to indicated in brackets e.g. 23% (23/100). This should be done all the data presented in this section. When reporting on the mutations, also indicate the amino acid or nucleotide change as well. For example, which mutation is present on codon 2058? Change from what to what?

Introduction:

- Second sentence, the authors to describe in detail the type of culture used to grow NTMs for 40 days. MGIT?
- Second paragraph, authors to indicate the duration of treatment of NTM infections. Also the length of treatment with Rifampicin to be mentioned. Is it the same for patients infected with comorbidities?
- The authors have listed several drugs used for the treatment of NTMs but described only one resistance mechanisms for NTMs, do they all have the same type of resistance mechanism? (Macrolides - Clarithromycon and Azithromycin).
- Paragraph seven, the authors to indicate that Rifampicin is recommended for first-line treatment of MTB alongside other drugs.

Laboratory procedures:

- The authors to estimate the volume of sputum sample collected from each participant.
- For the FM microscopy, how many readers were used before reporting the smear results?
- What volume of DNA was finally collected after extraction? Was quantification of the DNA material done?

Results:

- The authors to mention the table with results indicating that the bulk of the samples were obtained from Lake Victoria, Coastal and Nairobi region.

Discussion:

- The authors to refer to my previous comments on Non-tuberculous mycobacterium.
- The authors to critically discuss the relevance of the rrl significant mutations reported in the study sites. What are the implications of these finding?

Is the work clearly and accurately presented and does it cite the current literature?

Yes

If applicable, is the statistical analysis and its interpretation appropriate?

Yes

Are all the source data underlying the results available to ensure full reproducibility?

Yes

Is the study design appropriate and is the work technically sound?

Yes

Are the conclusions drawn adequately supported by the results?

Yes

Are sufficient details of methods and analysis provided to allow replication by others?

Yes

Reviewer Expertise:

TB molecular biology and immunology

I confirm that I have read this submission and believe that I have an appropriate level of expertise to confirm that it is of an acceptable scientific standard, however I have significant reservations, as outlined above.

# Response

**Published:** 2023-03-27

**Authors:** Zakayo Mwangi

**Affiliations:** Meru University of Science and Technology, Kenya

Thank you for your valuable feedback.

# Peer review: Reviewer response for version 4

**DOI:** 10.5256/f1000research.144441.r163993

**Published:** 2023-02-27

**Authors:** Leena Al-Hassan

**Affiliations:** 1 Department of Global Health and Infection, Brighton and Sussex Medical School, Brighton, UK

No further comments. Please see previous comments about the need to correlate findings with MIC.

Is the work clearly and accurately presented and does it cite the current literature?

Partly

If applicable, is the statistical analysis and its interpretation appropriate?

I cannot comment. A qualified statistician is required.

Are all the source data underlying the results available to ensure full reproducibility?

Partly

Is the study design appropriate and is the work technically sound?

Partly

Are the conclusions drawn adequately supported by the results?

Partly

Are sufficient details of methods and analysis provided to allow replication by others?

Partly

Reviewer Expertise:

Infectious diseases and antibiotic resistance

I confirm that I have read this submission and believe that I have an appropriate level of expertise to state that I do not consider it to be of an acceptable scientific standard, for reasons outlined above.

# Peer review: Reviewer response for version 3

**DOI:** 10.5256/f1000research.141647.r157361

**Published:** 2022-12-05

**Authors:** Leena Al-Hassan

**Affiliations:** 1 Department of Global Health and Infection, Brighton and Sussex Medical School, Brighton, UK

I have read the revised version, but I still see that the manuscript has misleading information that needs to be re-written. The data provided need to associated with phenotypic data on resistance. Identification of mutations in genes is not sufficient to prove resistance, as the researchers have not confirmed any silent mutations. 

 The positive controls used in the methodology should be isolates that are confirmed resistant and harbour these mutations. This is the definition of a positive control. The negative control is an isolates that is genotypically confirmed having no mutations in the target genes.

Is the work clearly and accurately presented and does it cite the current literature?

Partly

If applicable, is the statistical analysis and its interpretation appropriate?

I cannot comment. A qualified statistician is required.

Are all the source data underlying the results available to ensure full reproducibility?

Partly

Is the study design appropriate and is the work technically sound?

Partly

Are the conclusions drawn adequately supported by the results?

Partly

Are sufficient details of methods and analysis provided to allow replication by others?

Partly

Reviewer Expertise:

Infectious diseases and antibiotic resistance

I confirm that I have read this submission and believe that I have an appropriate level of expertise to state that I do not consider it to be of an acceptable scientific standard, for reasons outlined above.

# Response

**Published:** 2022-12-13

**Authors:** Zakayo Mwangi

**Affiliations:** Meru University of Science and Technology, Kenya

Thank you for the feedback.

# Peer review: Reviewer response for version 2

**DOI:** 10.5256/f1000research.139953.r154017

**Published:** 2022-11-22

**Authors:** Leena Al-Hassan

**Affiliations:** 1 Department of Global Health and Infection, Brighton and Sussex Medical School, Brighton, UK

Thank you for revising the manuscript and incorporating the comments into the text. However I would highly suggest that you revise the sections where you correlate the genotype with phenotype, since you have not done the AST (MIC) in this study. If you have not done the AST testing, you cannot state that they are resistant. Your study is primarily genotype based, and you have detected mutations in target resistance determinants, and I therefore suggest you change the wording of the results and discussion to reflect that. These mutations may be silent mutations, and if you do not have phenotype data to support it, you cannot state the fact that they are resistant. You can only say that they harbour the mutations which in previous reports have led to resistance. 

 These statements "Twenty-eight (23%) of the NTM were resistant to at least one of the antibiotics used in the macrolide-based treatment " and "Ten (14.7%) of the 68 SGM were resistant to rifampicin, with 40 percent having mutations at codon 531 in the rpoB gene" cannot be proven without AST. You should re-word it to say that 23% harboured mutations associated with resistance to macrolide antibiotics. 

 Same for the conclusion: you have not demonstrated a significant level of drug resistance for macrolides, aminoglycosides and rifampicin in NTM - you have detected mutations in gene targets that have previously been associated with drug resistance. 

 I hope my point is clear. I am not doubting your results, and I think it's interesting that you have detected these mutations, however it's not scientifically sound to assume resistance based on genotype only, and you must phrase your results and conclusions according to the methods used and data generated. The way it is written now indicates that you have the AST data for the isolates and you are sure that they are rifampicin or aminoglycoside resistant. I would strongly recommend you perform the AST - even for a subset of isolates - to confirm that the observed genotype correlates with the phenotype.

Is the work clearly and accurately presented and does it cite the current literature?

Partly

If applicable, is the statistical analysis and its interpretation appropriate?

I cannot comment. A qualified statistician is required.

Are all the source data underlying the results available to ensure full reproducibility?

Partly

Is the study design appropriate and is the work technically sound?

Partly

Are the conclusions drawn adequately supported by the results?

Partly

Are sufficient details of methods and analysis provided to allow replication by others?

Partly

Reviewer Expertise:

Infectious diseases and antibiotic resistance

I confirm that I have read this submission and believe that I have an appropriate level of expertise to state that I do not consider it to be of an acceptable scientific standard, for reasons outlined above.

# Response

**Published:** 2022-11-28

**Authors:** Zakayo Mwangi

**Affiliations:** Meru University of Science and Technology, Kenya

I appreciate your re-evaluation and the way in which you made the issues that need to be addressed evident.

 I revised the paper in accordance with your advice and resubmitted it for review.

# Peer review: Reviewer response for version 2

**DOI:** 10.5256/f1000research.139953.r154016

**Published:** 2022-11-03

**Authors:** Utpal Sengupta

**Affiliations:** 1 Stanley Browne Laboratory, The Leprosy Mission Community Hospital, New Delhi, Delhi, India

The manuscript can be accepted now.

Is the work clearly and accurately presented and does it cite the current literature?

Partly

If applicable, is the statistical analysis and its interpretation appropriate?

Yes

Are all the source data underlying the results available to ensure full reproducibility?

Yes

Is the study design appropriate and is the work technically sound?

Yes

Are the conclusions drawn adequately supported by the results?

Yes

Are sufficient details of methods and analysis provided to allow replication by others?

Yes

Reviewer Expertise:

Infectious diseases with special reference to tuberculosis and leprosy, Cell Biology, Microbiology, Immunology and Biotechnology.

I confirm that I have read this submission and believe that I have an appropriate level of expertise to confirm that it is of an acceptable scientific standard.

# Response

**Published:** 2022-11-28

**Authors:** Zakayo Mwangi

**Affiliations:** Meru University of Science and Technology, Kenya

We sincerely appreciate your second reading and approval.

# Peer review: Reviewer response for version 1

**DOI:** 10.5256/f1000research.136168.r150334

**Published:** 2022-10-10

**Authors:** Utpal Sengupta

**Affiliations:** 1 Stanley Browne Laboratory, The Leprosy Mission Community Hospital, New Delhi, Delhi, India

The manuscript has been written on the findings of antimycobacterial resistance of NTM isolated from 122 sputum *M. tuberculosis*-negative patients. The authors have investigated their resistance patterns on the isolated strains using gene sequencing of the drug-resistant genes and noted the mutations in the respective codon positions.

 Materials and methods have been well described. Data were properly analyzed and presented adequately in the tables. However, there were a lot of discrepancies in Table 2 in the first column where the total sample number was shown to be 135 instead of 122 and therefore the percentage expressions were wrongly mentioned in the table and in the text. The percentages have to be calculated correctly and Table 2 has to be modified and described accordingly in the text.

 The discussion has been adequate, citing adequate references. However, the discussion with respect to the results of Table 2 has to be modified and discussed for a correct understanding of the reader.

Is the work clearly and accurately presented and does it cite the current literature?

Partly

If applicable, is the statistical analysis and its interpretation appropriate?

Yes

Are all the source data underlying the results available to ensure full reproducibility?

Yes

Is the study design appropriate and is the work technically sound?

Yes

Are the conclusions drawn adequately supported by the results?

Yes

Are sufficient details of methods and analysis provided to allow replication by others?

Yes

Reviewer Expertise:

Infectious diseases with special reference to tuberculosis and leprosy, Cell Biology, Microbiology, Immunology and Biotechnology.

I confirm that I have read this submission and believe that I have an appropriate level of expertise to confirm that it is of an acceptable scientific standard, however I have significant reservations, as outlined above.

# Response

**Published:** 2022-10-17

**Authors:** Zakayo Mwangi

**Affiliations:** Meru University of Science and Technology, Kenya

I appreciate your review. The issue mentioned above has been addressed.

# Peer review: Reviewer response for version 1

**DOI:** 10.5256/f1000research.136168.r148288

**Published:** 2022-09-22

**Authors:** Leena Al-Hassan

**Affiliations:** 1 Department of Global Health and Infection, Brighton and Sussex Medical School, Brighton, UK

This study characterises mutation patterns for resistance genes in NTM from Kenya. 

 Although the study is interesting, I think the authors need to give more information in the methodology and results section on:

- Identification: 122 isolates were collected, but no details in the results on how many were slow-growing and rapid-growing NTM. 
- MIC: No mention of methodology for MIC and results.
- The PCR: Did you include any positive or negative controls in the experiments?
- The discussion on mutations and their role in resistance is quite vague. It's not clear whether the mutations identified can be linked to the observed MIC. Have the researchers checked that these are not silent mutations?

Is the work clearly and accurately presented and does it cite the current literature?

Partly

If applicable, is the statistical analysis and its interpretation appropriate?

I cannot comment. A qualified statistician is required.

Are all the source data underlying the results available to ensure full reproducibility?

Partly

Is the study design appropriate and is the work technically sound?

Partly

Are the conclusions drawn adequately supported by the results?

Partly

Are sufficient details of methods and analysis provided to allow replication by others?

Partly

Reviewer Expertise:

Infectious diseases and antibiotic resistance

I confirm that I have read this submission and believe that I have an appropriate level of expertise to confirm that it is of an acceptable scientific standard, however I have significant reservations, as outlined above.

# Response

**Published:** 2022-09-27

**Authors:** Zakayo Mwangi

**Affiliations:** Meru University of Science and Technology, Kenya

I appreciate your review.

- Under methods, it has been indicated that of the 122 NTM isolated, 54 were rapid-growing NTM while 68 were slow-growing NTM.
- The phenotypic drug sensitivity by MIC analysis was not done in this study.  We performed genotypic characterization of the drug target genes by describing the mutations associated with drug resistance through target sequencing of the drug target genes.
- Positive and negative controls were included during PCR and subsequent sequencing of respective target genes. The controls were based on Genotype NTM-DR results.
- In the discussion, the mutations identified in this study have been compared to those seen in previous studies that had also included MIC in their analysis. The point mutations identified in the drug target genes were seen to be associated with phenotypic drug resistance.
